# HALP score based on hemoglobin, albumin, lymphocyte and platelet can predict the prognosis of tongue squamous cell carcinoma patients

**DOI:** 10.1016/j.heliyon.2023.e20126

**Published:** 2023-09-13

**Authors:** Dandan Zhang, Shan Chen, Wei Cao, Ningbo Geng, Chongjin Feng

**Affiliations:** Department of Stomatology, The Frist Affiliated Hospital of Sun Yat-Sen University, Guangzhou, Guangdong, 510080, People's Republic of China

**Keywords:** HALP (Hemoglobin Albumin Lymphocyte And Platelet) score, Tongue squamous cell carcinoma, Prognosis, Immune, Inflammation

## Abstract

**Objective:**

The preoperative hemoglobin, albumin, lymphocyte, and platelet (HALP) score, a comprehensive marker of nutritional and immunological status, has been found to be robust for tumor prognosis prediction. Here, we evaluated the use of HALP in the prognostic prediction of tongue squamous cell carcinoma (TSCC).

**Study design:**

Patients with TSCC were retrospectively recruited from the years 2009–2019. Patient clinicopathological characteristics, along with preoperative blood parameters, were recorded on admission, and the cut-off HALP value was determined by X-tile software. Kaplan-Meier curves and Cox regression analyses were used to evaluate the predictive value of HALP for patient overall survival (OS) and disease-free survival (DFS).

**Results:**

A total of 339 TSCC patients were enrolled. The optimal HALP threshold was 56 and the patients were divided into two groups according to their scores. The Kaplan-Meier analysis showed that patients in the high-HALP group experienced longer OS (*p =* 0.007) and DFS (*p* = 0.006) than those in the low-HALP group. Multivariate analysis showed that elevated HALP (*p =* 0.038) was an independent predictor of OS, while age (*p =* 0.008), T stage (*p* < 0.001), N stage (*p* = 0.020), and degree of tumor differentiation (p < 0.001) were risk factors.

**Conclusion:**

The findings showed that the preoperative HALP score was an independent predictor of prognosis in patients with TSCC.

## 1 Introduction

Cancers of the lip and oral cavity rank ninth in prevalence in males and 15th in females [1], with tongue squamous cell carcinoma (TSCC) accounting for approximately 40% [2] and thus representing the most common form of oral cancer. Unfortunately, TSCC is prone to cervical lymph node metastasis due to frequent tongue movements, as well as the presence of abundant lymphatic vessels and neurovascular bundles in the head and neck regions [3]. Moreover, the tumor is often not diagnosed at an early stage [4], and combined with its tendency for occult lymph node metastasis [5], patient prognosis is extremely poor. Statistically, only 50–60% of TSCC patients survive for more than five years from the time of diagnosis [6].

Various biomarkers associated with TSCC prognosis have been identified. However, most of these, including TNM staging, are based on postoperative pathological results. Hence, it is both urgent and necessary to identify preoperative indicators for TSCC patients as early detection can enhance the personalized care of patients, thus significantly improving outcomes. Multiple reports have confirmed that certain preoperative indicators of nutritional status, such as albumin [7], and derived indicators such as the prognostic nutritional index (PNI), are critical prognostic indicators for various solid tumors [8,9]. Additionally, the hemoglobin, albumin, lymphocyte, and platelet (HALP) score is a promising indicator of both nutritional and inflammatory status, and its prognostic value has recently been confirmed in hepatocellular carcinoma, gastric cancer, colorectal cancer, and small cell lung cancer [[10], [11], [12], [13]]. Nevertheless, there is limited information on the prognostic significance of the HALP score in head and neck cancer. Moreover, its association with the prognosis of patients with tongue cancer is yet to be determined.

The tongue serves a critical function in chewing and other aspects. The progression of tongue cancer often involves deep muscles or surrounding tissues, which ultimately restrict tongue movement, thereby limiting speech, chewing, swallowing, and other functions. The current mainstay of TSCC treatment is tumor resection, as well as appropriate neck dissection accompanied by radiotherapy and chemotherapy. However, the patient's condition may be aggravated by the surgery, due to postoperative complications such as mastication efficiency, dysphagia, and other dysfunctions, resulting in insufficient nutritional intake. Hence, the nutritional status of TSCC patients is closely associated with prognosis. In particular, malnutrition in tongue cancer patients is indicative of poor prognosis. As a comprehensive bioindicator, HALP not only represents the nutritional indices of hemoglobin and albumin but also the immune indices of lymphocyte and platelet counts, and is thus likely to be more accurate in the prediction of prognosis in patients with tongue cancer than a single index. Considering this evidence, the present study aimed to evaluate the role of HALP as an independent predictor of prognosis in patients with TSCC.

## 2 Materials and methods

### 2.1 Patients and treatment

The study was approved by the ethics committee of the First Affiliated Hospital of Sun Yat-sen University and was conducted in compliance with the most recent version of the Declaration of Helsinki. We retrospectively collected and examined data from 339 TSCC patients who were treated at the hospital between January 2009 and December 2019. All included patients before 2018 were treated using the 2009 version of the NCCN guidelines, while patients after 2018 were treated using the 2018 version of the guidelines. All participants underwent surgical tumor resection. Moreover, based on published evidence, cervical lymph node dissection, as well as radiotherapy and chemotherapy, were performed, as appropriate. The inclusion criteria were: (1) A pathological diagnosis of TSCC; (2) Absence of distant metastasis; (3) No new adjuvant treatment performed before radical resection. The exclusion criteria were: (1) Presence of other malignant tumors; (2) Evidence of severe infection before surgery; (3) Presence of autoimmune disease; (4) Patient suffering from hematological diseases; (5) Missing follow-up information.

### 2.2 Data acquisition and patient follow-up

We collected the baseline patient information and clinicopathological data, including information on sex, age, smoking status, depth of invasion, tumor size, and degree of differentiation. The hematological parameters included leukocyte, neutrophil, lymphocyte, and platelet counts, as well as the levels of hemoglobin, albumin, and globulin. The HALP score was calculated using the following formula: Hemoglobin (g/L) × Albumin (g/L) × Lymphocyte (/L)/Platelet (/L). For patients who were treated before the release of the 8th edition of the AJCC TNM classification, a reclassification was performed so that all patient staging was based on the 8th edition of the AJCC.

Overall survival (OS) was defined as the interval between the date of surgery and the death or last follow-up assessment of the patient. Disease-free survival (DFS) was defined as the interval between the date of surgery and the diagnosis of locoregional recurrence or metastasis, or the last follow-up. Postoperative telephonic follow-ups were conducted every three months for the first two years, every six months for the following two years, then annually until disease recurrence or death. Recurrence and metastasis were further confirmed using computed tomography (CT), magnetic resonance imaging (MRI), positron emission tomography/computed tomography (PET/CT), or pathological reports from re-surgery. The last follow-up was on October 31, 2021.

### 2.3 Statistical analysis

Categorical variables are presented as frequencies and percentages while continuous variables are presented as means ± standard deviation. Differences in categorical variables between the HALP groups were assessed using Pearson Chi-square tests. The optimal cut-off for HALP scoring was determined using X-tile software. Briefly, each HALP score was used as the cut-off value for the log-rank test, and the value with the lowest p-value was selected as the optimal cutoff value. Kaplan-Meier curves for used for survival analysis. Significant differences in survival rates associated with prognostic indicators were assessed by log-rank tests. Univariate and multivariate Cox regression analyses were used to determine the prognostic significance of hematological parameters while adjusting for confounding factors. Proportional hazards analysis was performed before the multivariate analysis. For the continuous variable of age, Schoenfeld residuals was test used to validate the assumption and other Categorical variables, we used the Kaplan-Meier survival curve to verify proportional hazards assumption by observing whether there is a crossover in the survival curve. The results demonstrated that all categorical variables met the necessary conditions. Hazard ratios (HR) and 95% confidence intervals (95% CI) are provided for both the univariate and multivariate analyses. Variables that were found to have prognostic significance for OS in the Cox regression analysis were then used for the construction of a nomogram for prognosis prediction using R 4.2.3 software [14]. The concordance index (C-index) was used to evaluate the discrimination of the nomogram. Calibration plots were used to assess the predictive performance of the nomogram. All data were analyzed using SPSS 25.0 and X-tile v3.6.1 (Yale University) software. Two-tailed p < 0.05 was set as the significance threshold.

## 3 Results

### 3.1 Baseline characteristics

We retrieved the baseline clinicopathological characteristics of all TSCC patients in the cohort. As summarized in Table 1, patients with TSCC were selected for analysis, including 210 (61.9%) males and 129 (38.1%) females, with a mean age of 57.4 years (range: 18–96 years) and 133 (39.2%) of whom were smokers. Additionally, 100 (29.5%), 105 (31.0%), 66 (19.5%), and 68 (20.1%) patients were classified as having stage I, II, III, and IV disease, respectively. Lymph node metastasis was present in 106 (31.3%) patients. We next divided the patients into three groups according to the degree of tumor differentiation as follows: 122 (36.0%), well-differentiated; 167 (49.3%) moderately differentiated; 50 (14.7%) poorly differentiated. The infiltration depth was less than 5 mm in 156 (46%) cases, 5–10 mm in 84 (24.8%) cases, and greater than 10 mm in 99 (29.2%) cases. Lastly, the median follow-up duration was 39 months (range：3–152 months).

**Table 1:** Clinicopathological characteristics of patients with tongue squamous cell carcinoma.

Variables(n = 339)	n(%)
Age(yrs)	
mean ± SD	57.4(18–96)
**Gender**	
Male	210 (61.9)
Female	129 (38.1)
**Smoking**	
Yes	133 (39.2)
No	206 (60.8)
**Drinking**	
Yes	103(30.4)
No	236(69.6)
**T Staging**	
T1	112 (33.0)
T2	150 (44.2)
T3	68 (20.1)
T4	9 (2.7)
**N Staging**	
N0	233 (68.7)
N+	106 (31.3)
**Stage**	
I	100 (29.5)
II	105 (31.0)
III	66 (19.5)
IV	68 (20.1)
**Differentiation degree**	
Well	122 (36.0)
Moderate	167 (49.3)
Poor	50 (14.7)
**Infiltration depth(mm)**	
≤5	156 (46.0)
5-10	84 (24.8)
≥10	99 (29.2)
**Margin status(mm)**	
≤5	41(12.1)
>5	298(87.9)
**HALP**	
Median(interquartile range)	52.49(39.29–68.64)

HALP: hemoglobin, albumin, lymphocyte and platelet.

### 3.2 Threshold for hematological parameters

**Fig. 1:**
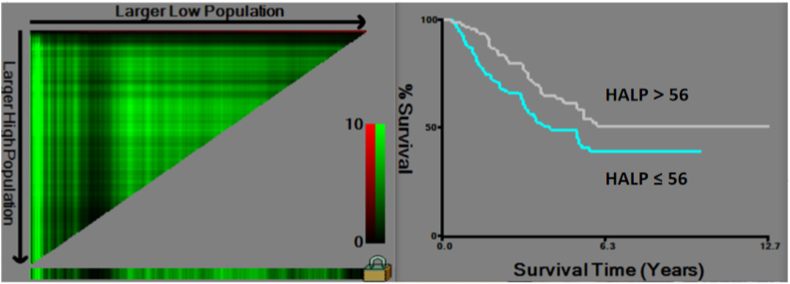
Cut-off value of HALP by X-tile software. HALP, hemoglobin, albumin, lymphocyte and platelet.

The cut-off value of HALP was 56. X-tile software was used to determine the optimal threshold for the pretreatment HALP score. This is a tool for biomarker assessment and cut-point optimization. Based on X-tile, the optimal HALP score was set at 56 (Fig. 1).

### 3.3 Clinical characteristics of TSCC patients in the different groups

**Fig. 2:**
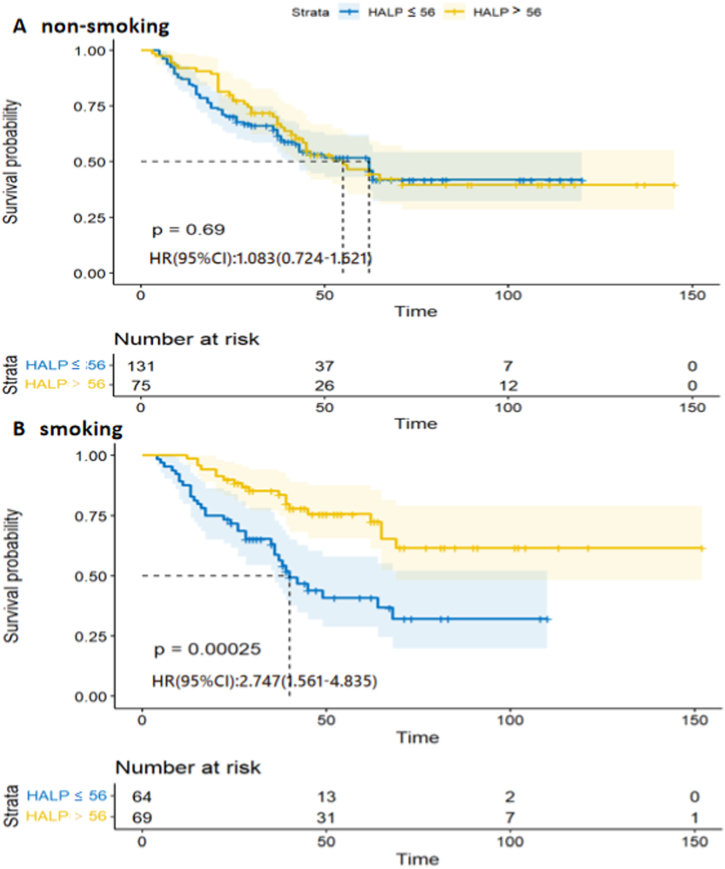
The Kaplan-Meier survival curves stratified by smoking status and grouped by HALP score. A Overall survival of non-smoking group B Overall survival of smoking group. HALP, hemoglobin, albumin, lymphocyte, and platelet.

The HALP score was found to be associated with patient sex, smoking status, and lymph node metastasis. The study participants were assigned to two groups according to the HALP cut-off score of 56. Accordingly, there were 144 patients in the elevated HALP cohort and 195 patients in the reduced HALP cohort. The differences in baseline characteristics between the two groups were then assessed by Chi-square tests. It was found that HALP scores were significantly associated with both patient sex (*p* < 0.001) and smoking status (*p* < 0.001). As the HALP score varied with smoking status, we divided patients into two groups based on whether they smoked or not. We performed further analysis to explore the predictive value of HALP in different smoking groups. This showed that patients in the low-HALP group had shorter OS than patients in the high-HALP group who smoked (*p* < 0.001) (Fig. 2B). There was no significant difference in OS between the high- and low-HALP groups in patients who did not smoke (Fig. 2A). Additionally, patients in the low-HALP group were more likely to develop lymph node metastasis (*p* = 0.002), relative to patients in the high-HALP cohort (Table 2).

**Table 2:** Clinicopathological characteristics in different HALP score groups.

Variables(n = 339)	**HALP**		P
	≤56(n, %)	>56(n, %)	
**Age(yrs)**			
≤60	107(54.9)	80(55.6)	0.900
>60	88(45.1)	64(44.4)	
**Gender**			
Male	109(55.9)	101(70.1)	**0.008**
Female	86(44.1)	43(29.9)	
**Smoking**			
Yes	64(32.8)	69(47.9)	**0.005**
No	131(67.2)	75(52.1)	
**Drinking**			
Yes	53(27.2)	50(34.7)	0.136
No	142(72.8)	94(65.3)	
**T Staging**			
T1-T2	154(79.0)	108(75.0)	0.388
T3-T4	41(21.0)	36(25.0)	
**N Staging**			
N0	121(62.1)	112(77.8)	**0.002**
N+	74(37.9)	32(22.2)	
**Differentiation degree**			
Well	63(32.3)	59(41.0)	0.182
Moderate	99(50.8)	68(47.2)	
Poor	33(16.9)	17(11.8)	
**Infiltration depth(mm)**			
≤10	159(81.5)	110(76.4)	0.247
>10	36(18.5)	34(23.6)	
**Margin status(mm)**			
≤5	29(14.9)	12(8.3)	0.068
>5	166(85.1)	132(91.7)	

HALP: hemoglobin, albumin, lymphocyte and platelet.

### 3.4 Association between hematological parameters and patient OS and DFS

The HALP score, differentiation degree, T staging, and N staging were found to be independent prognostic factors for both OS and DFS. The clinicopathological features and hematological indicators of patients were then included in the univariate and multivariate Cox proportional hazards analyses to determine their prognostic significance in estimating patient OS and DFS. The univariate analysis showed that patient OS was related to age (*p* = 0.010), T stage (*p <* 0.001), N stage (*p <* 0.001), degree of tumor differentiation (*p <* 0.001), and the HALP score (*p* = 0.007) (Table 3). Moreover, the univariate analysis also revealed that the DFS was associated with T stage (*p <* 0.001), N stage (*p <* 0.001), degree of tumor differentiation (*p <* 0.001), and the HALP score (*p* = 0.006) (Table 4). Thus, patients with low HALP scores, poor tumor differentiation, and higher T and N stages were significantly more likely to experience worse OS and DFS. Based on our observations, neither OS nor DFS was related to sex.

**Table 3:** Univariate and multivariate analysis of overall survival in the cohort of 339 TSCC patients.

Variable	Univariate analysis		Multivariate analysis	
	HR(95％CI)	p	HR(95％CI)	p
**Age**	1.014(1.003–1.025)	**0.010**	1.016(1.004–1.027)	**0.008**
**Gender**				
Male	1	0.754	1	0.527
Female	0.949(0.686–1.314)		0.893(0.629–1.268)	
**Smoking**				
Yes	1	0.133		
No	1.290(0.925–1.799)			
**Drinking**				
Yes	1	0.840		
No	1.036(0.738–1.455)			
**HALP**				
≤56	1	**0.007**	1	**0.038**
>56	0.639(0.460–0.887)		0.693(0.489–0.981)	
**T Staging**				
T1- T2	1	**<0.001**	1	**<0.001**
T3- T4	2.790(1.991–3.909)		2.543(1.711–3.780)	
**N Staging**				
N0	1	**<0.001**	1	**0.020**
N+	2.313(1.673–3.197)		1.555(1.073–2.254)	
**Differentiation degree**				
Well	1		1	
Moderate	1.531(1.050–2.233)	**0.027**	1.428(0.975–2.091)	0.067
Poor	3.447(2.203–5.392)	**<0.001**	2.965(1.876–4.685)	**<0.001**
**Margin status(mm)**				
≤5	1	0.443		
>5	0.832(0.520–1.332)			

HALP: hemoglobin, albumin, lymphocyte and platelet. HR hazard ratio, CI confidence interval.

**Table 4:** Univariate and multivariate analysis of disease-free survival in the 339 TSCC patients.

Variable	Univariate analysis		Multivariate analysis	
	HR(95％CI)	p	HR(95％CI)	p
Age	1.001(0.991–1.012)	0.805	1.002(0.992–1.013)	0.678
Gender				
**Male**	1	0.841	1	0.861
**Female**	1.033(0.749–1.426)		0.970(0.687–1.369)	
Smoking				
Yes	1	0.459		
No	1.128(0.820–1.554)			
Drinking				
Yes	1	0.444		
No	1.137(0.818–1.582)			
HALP				
**≤**56	1	**0.006**	1	**0.047**
>56	0.637(0.460–0.881)		0.707(0.502–0.995)	
T Staging				
**T1- T2**	1	**＜0.001**	1	**0.001**
**T3- T4**	2.349(1.681–3.284)		1.959(1.327–2.892)	
N Staging				
**N0**	1	**＜0.001**	1	**0.012**
**N+**	2.272(1.655–3.117)		1.599(1.110–2.302)	
Differentiation degree				
**Well**	1		1	
**Moderate**	1.449(1.008–2.081)	**0.045**	1.267(0.876–1.833)	0.209
**Poor**	2.357(1.498–3.709)	**＜0.001**	1.850(1.158–2.955)	**0.010**
Margin status(mm)				
≤5	1	**0.063**		
>5	0.662(0.428–1.023)			

HALP: hemoglobin, albumin, lymphocyte and platelet. HR hazard ratio, CI confidence interval.

**Fig. 3:**
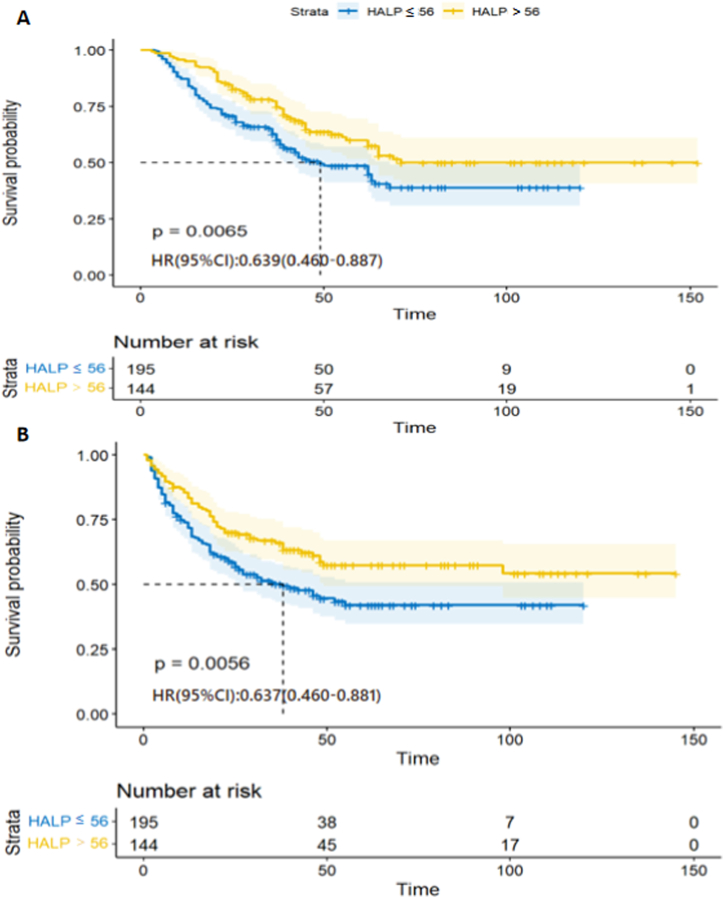
The survival curves grouped by HALP score with the Kaplan-Meier analysis. A Overall survival B Disease-free survival. HALP, hemoglobin, albumin, lymphocyte, and platelet.

Kaplan-Meier curves and log-rank tests showed strong associations between HALP and OS and DFS (*p* = 0.007 and *p* = 0.006, respectively). Specifically, patients in the low-HALP group were significantly more likely to have reduced experience OS (Fig. 3A) and DFS (Fig. 3B).

We next adjusted the risk factors by selecting the significant indicators from the univariate analysis and entering them into the multivariate Cox regression analysis. This showed that low HALP score (*p* = 0.038), high T stage (*p <* 0.001), high N stage (*p* = 0.020), poor tumor differentiation (*p <* 0.001), and older age (*p* = 0.008) were significant independent predictors of reduced OS (Table 3). Additionally, low HALP scores (*p* = 0.047), poor tumor differentiation (*p* = 0.010), high T stage (*p* = 0.001), and high N stage (*p* = 0.012) were found to be independent predictors of worse DFS (Table 4).

### 3.5 Nomogram for OS

**Fig. 4:**
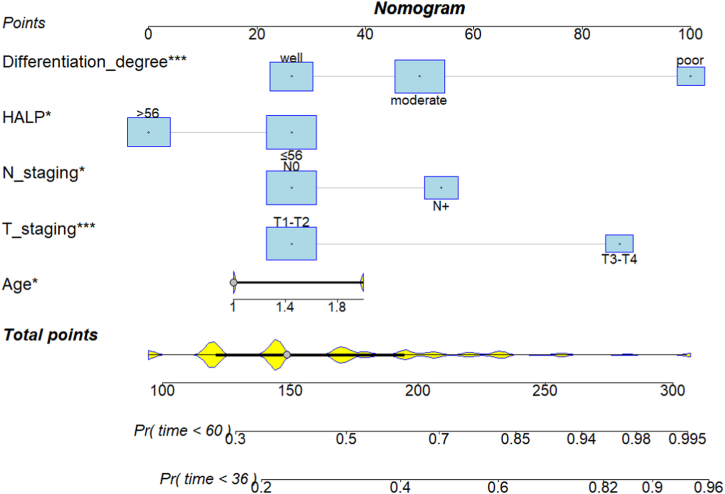
A prognostic nomogram model based on age, T staging, N staging, tumor differentiation degree and HALP of tongue squamous cell carcinoma. HALP, hemoglobin, albumin, lymphocyte, and platelet.

**Fig. 5:**
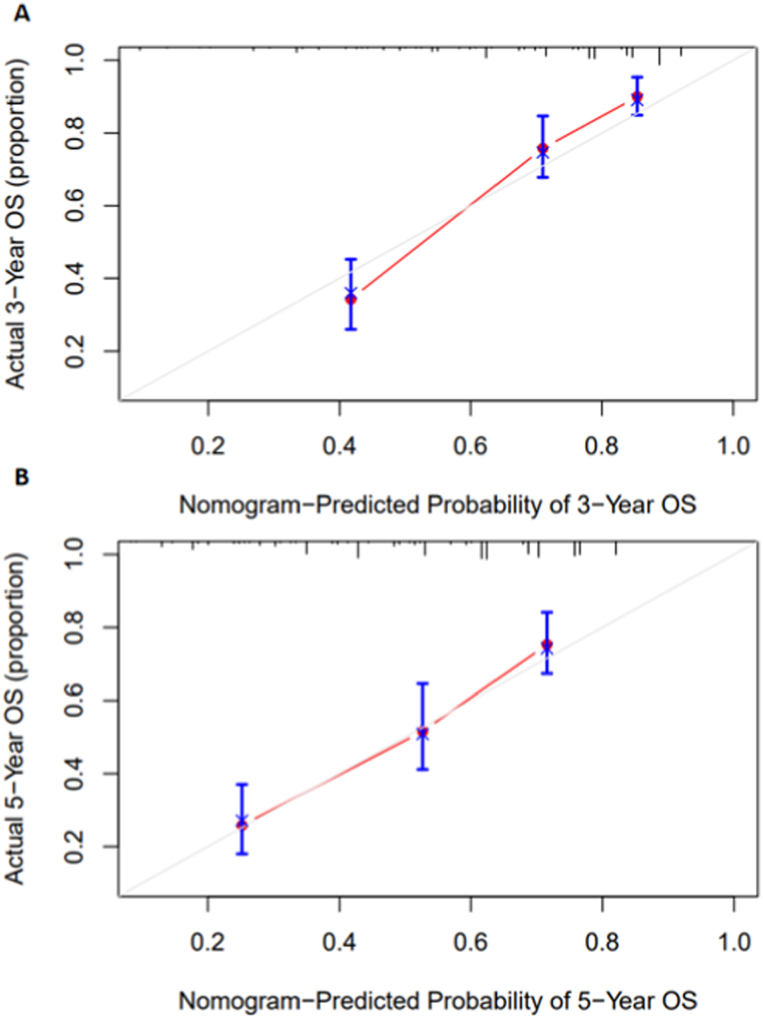
Calibration plots for the nomogram model. A 3-year overall survival B 5-year overall survival.

To visualize the results of the Cox regression analysis, and to evaluate the predictive significance of factors such as HALP on TSCC prognosis, we next generated a nomogram model using the significant prognostic indicators from the multivariate analysis, namely, age, T stage, N stage, degree of tumor differentiation, and HALP score (Fig. 4). The concordance index (CI) of the nomogram was 0.731. We further developed a nomogram calibration plot that describes the consistency between the 3-year (Fig. 5A) and 5-year (Fig. 5B) predicted and the observed survival probability, and the results indicated good predictive performance.

## 4 Discussion

Univariate regression analysis showed that TSCC patients with reduced HALP scores exhibited significantly worse OS and DFS. This study is the first to report the use of the HALP score as an independent prognostic indicator in TSCC. The multivariate analysis further showed that patient age, T stage, N stage, and degree of tumor differentiation were independent predictors of prognosis.

In recent years, the association between preoperative peripheral blood parameters and tumor prognosis has been intensively investigated. Patient prognosis has been shown to be strongly associated with tumor invasiveness as well as the host anti-tumor immunoinflammatory status [14,15]. HALP is a parameter that is able to represent both nutritional and inflammatory status and the HALP score has recently been reported to be associated with the prognosis of multiple tumors. HALP is calculated as follows: hemoglobin (g/L) × Albumin (g/L) × Lymphocytes (/L)/Platelets (/L), and it was first proposed by Chen in 2015, who reported HALP to be a stand-alone predictor of gastric cancer [16]. One study involving 319 patients with pharyngeal cancer identified HALP as a stand-alone prognostic factor for OS and cancer-specific survival (CSS). It is worth mentioning that in this study, the authors combined other indicators such as prognostic nutritional index (PNI), nutritional risk index (NRI), and body mass index (BMI) with HALP to predict the prognosis of pharyngeal cancer [17]. In another study, involving 1580 patients with early cervical cancer, HALP was reported to be a stand-alone predictor of cervical cancer recurrence [18]. The study had a sufficiently large sample size and higher reliability.

Although the prognostic value of HALP has been confirmed in various tumors, the use of the HALP score still has its drawbacks. Firstly, since HALP is a composite indicator calculated from four indicators, it varies with the changes in the individual indicators. The presence of severe infections and autoimmune disease will have a significant impact on the lymphocyte count; thus, we excluded these patients from our study. Secondly, the underlying mechanism by which HALP affects prognosis is still unclear. Thirdly, in current research, HALP does not have a standardized cutoff value, which may lead to heterogeneity between different studies.

Nevertheless, the prognostic value of HALP is still worth further research. There are no reports on the possible correlation between HALP and TSCC prognosis. Thus, this study postulated that HALP may also have prognostic value in TSCC.

The tongue is a critical functional organ in the oral cavity and is required for chewing, speech, and taste. TSCC accounts for the highest proportion of oral cancers and is considerably more invasive. Following surgical resection, the tongue can become dysfunctional, with reduced mastication efficiency and dysphagia, which can result in insufficient nutritional intake and reduced hemoglobin. The prognosis of TSCC is relatively poor; hence, it is crucial to identify independent prognostic indicators such as HALP for the early intervention of oral cancer, quick and efficient treatment, and improved prognosis. HALP is composed of four indicators of which hemoglobin and albumin are the most closely related to nutritional status, while lymphocytes represent an important part of the immune system. These reasons are potentially why HALP influences TSCC prognosis. A more precise mechanism may be explained in terms of the effects of the four components of HALP.

The hemoglobin level is a robust indicator of oxygen transport. Reduced hemoglobin concentration is generally associated with poor tumor prognosis [19]. A decline in hemoglobin may result from malnutrition, increased bleeding within solid tumors of advanced cancer patients, and reduced myelosuppressive erythrocyte synthesis resulting from chemotherapy drugs [20]. Reduced hemoglobin levels lead to insufficient oxygen transport. In an anoxic environment, tumor neovascularization is enhanced by the activation of angiogenic factors and chemokines which, in turn, accelerates tumor invasion and metastasis. Hypoxia not only promotes tumor cell immune escape through the induction of programmed cell death but also regulates metabolism by aggregating lactic acid in the tumor microenvironment, thereby enhancing immune cellular stress and tumor immune tolerance. Moreover, hypoxia promotes the epithelial-mesenchymal transition, which leads to tumor progression [21].

Serum albumin levels are also strongly associated with nutritional status. Albumin, a liver byproduct, typically makes up over 50% of the entire plasma protein content [22], and it is considered a negative acute phase protein. Albumin levels may be reduced by varying degrees due to malnutrition, such as advanced cachexia, as well as inhibition of inflammatory reactions, resulting in poor prognosis in cancer patients. It also has a confirmed prognostic role in breast [23] and endometrial cancers [24]. Insufficient postoperative nutritional intake in TSCC patients is known to lower both hemoglobin and albumin concentrations.

Furthermore, lymphocytes, the immune cells responsible for antitumor effects, enhance cancer patient prognosis by inhibiting tumor cell proliferation, migration, and metastasis [25,26].

It is reported that peripheral blood lymphocyte depletion results in diminished immune function and poor prognosis, which has been found in colorectal cancer [27] and other malignancies. Previous studies have shown that the platelet level is negatively associated with many aspects of tumor biology, including tumor proliferation, invasion, and metastasis. Platelets not only release vascular endothelial growth factor (VEGF) to enhance tumor angiogenesis [28], but also promote the secretion of tumor growth factor β(TGF-β), which reduces the functions of natural killer cells, leading to increased proliferation of cancer cells [29].

The HALP score integrates the functions of these four components, reflecting the immune, nutritional, and inflammatory status of patients. In this study, the threshold value for HALP was determined to be 56, which differed from another study examining 401 patients with non-small cell lung cancer, where the critical value was found to be 23.24 [11]. Likewise, in a study involving 582 patients with pancreatic cancer, the cut-off value was 44.56 [12],which was obtained using X-tile software, similar to the tool used in this study. These discrepancies in the optimal cut-off values may be associated with individual differences associated with the various studies. Based on our research, the HALP score was significantly correlated with patient sex, smoking status, and lymph node metastasis. Thus, HALP, an easily measured indicator, is a potent predictor of TSCC prognosis and does not cause additional trauma and economic burden to the patient.

In addition, the nomogram established in this study demonstrated good discriminative ability and accuracy. Compared with the traditional rough estimation of patient prognosis based on TNM staging, this model can predict the 3-year and 5-year survival rates of TSCC patients.

Although this study explored meaningful challenges, it has several limitations. First, this was a retrospective study spanning over 10 years. Due to significant advances in the measurement of indicators and the treatment of cancer, bias in data selection and heterogeneity of the included subjects are inevitable. As in this study, the absence of data on perineurial invasion, lymphovascular invasion and bone invasion is the main limitation. Thus, further large-scale prospective studies are needed to verify the conclusions of the present study. Secondly, HALP, as a composite indicator, is prone to change with each component and thus has some sensitivity and instability. Thirdly, the cut-off value of HALP varies greatly between different studies, including ours. In this study, the cut-off value of HALP was 56, which requires further examination in a study with a larger sample size.

## 5 Conclusion

Here, we demonstrated that the HALP score is a promising independent predictor of outcomes in patients with TSCC. Thus, the inclusion of HALP in the clinical prognostic evaluation system for tumors may contribute positively to the risk stratification of TSCC patients.

## Ethics approval and consent to participate

The study was authorized by the Medical Ethics Committee of the Frist Affiliated Hospital of Sun Yat-Sen University (Project No. [2022]045) and was conducted in compliance with the most recent version of Helsinki Declaration. Consent to Participate was not required for this retrospective study.

## Fundings

This research did not receive any specific grant from funding agencies in the public, commercial, or not-for-profit sectors.

## Author contribution statement

Chongjin Feng: Conceived and designed the experiments; Contributed reagents, materials, analysis tools or data. Dandan Zhang: Conceived and designed the experiments; Performed the experiments; Analyzed and interpreted the data; Contributed reagents, materials, analysis tools or data; Wrote the paper. Shan Chen: Analyzed and interpreted the data; Contributed reagents, materials, analysis tools or data; Wrote the paper. Wei Cao: Contributed reagents, materials, analysis tools or data. Ningbo Geng: Analyzed and interpreted the data.

## Data availability statement

Data will be made available on request.

## Declaration of competing interest

The authors declare that they have no known competing financial interests or personal relationships that could have appeared to influence the work reported in this paper.
